# Supplementary material for: Are carbon emissions trading and green financial instruments synergistic? -Comprehensive quantitative research based on content analysis
Source: PLoS One. 2024 Mar 7;19(3):e0298601. doi: 10.1371/journal.pone.0298601 (PMC10919601; doi:10.1371/journal.pone.0298601)
Supplement: S1 Data — (ZIP) [file pone.0298601.s001.zip › Data/地级市Z_F工作报告.zip!/╘╞─╧/▒ú╔╜╩╨/└┤╫╘╣1⁄2╓┌║┼í╛┬φ┐╦╩2╛▌═°í┐_2016.docx]

各位代表：

现在，我代表市人民政府，向大会报告政府工作。请各位代表连同《保山市国民经济和社会发展第十三个五年规划纲要（草案）》一并审议，请市政协委员和列席人员提出意见。

一、同心协力、攻坚克难，“十二五”发展创辉煌

2015年，市人民政府团结带领全市各族人民，深入学习贯彻习近平总书记系列重要讲话和考察云南指示精神，认真落实党中央国务院和省委省政府、市委的各项决策部署，扎实开展“实现新跨越，争当排头兵”大讨论活动，着力稳增长、促改革、调结构、惠民生，经济社会保持平稳较快发展。实现生产总值552亿元，同比增11.5%，居全省首位；固定资产投资501.8亿元，增30.7%；引进市外到位资金546亿元，增32.4%；社会消费品零售总额178.5亿元，增12.5%；一般公共预算收入52.2亿元，增10.7%；一般公共预算支出192亿元，增17%；金融机构存贷款余额775亿元和550.9亿元，分别增21.4%和23.1%；居民消费价格指数102.1%。全省上半年工作汇报暨园区建设等重要会议在我市召开，保山工作得到省委省政府充分肯定。除进出口总额和城镇常住居民可支配收入外，年初确定的目标任务均全面完成。

一年来，我们主要抓了以下工作：

——调结构、转方式，不断强化产业培育。实现农业增加值142亿元，增6.5%，粮食总产143.2万吨，增2万吨。收购烤烟128.2万担、香料烟24.5万担，质量效益稳定增长。实现畜牧业产值98亿元，增8.7%。新增省级重点农业龙头企业6户、市级农业产业化龙头企业32户，认定市级示范家庭农场50个，名牌农产品33个。轻纺、大数据等产业发展迈出坚实步伐，实现工业增加值135.9亿元，增14.1%。商贸、金融等服务业持续发展，火山热海5A级景区创建通过国家验收，全市接待海内外旅游者1280万人次，增16.2%，实现旅游业总收入112.8亿元，增28.4%。

——扩投资、强基础,不断增强发展后劲。保泸高速公路开工建设，大瑞铁路、绕城高速公路等项目建设进展顺利，大链二级公路试通车；工业园区配套设施不断完善，龙陵动力锂离子电池生产线、保山国际数据产业园开工，宝佳鞋业、九隆酵母建成投产；中心城市地下综合管廊、棚户区改造、“三校一院”建设加快推进，永昌路等一批老城市政道路完成改造提升，城市综合整治成效明显；各县城持续扩容提质，城乡统筹发展试点扎实推进，腾冲创建国家园林县城通过验收；新开工保山小地方中型水库和昌宁桂花树等5座小型水库；建成蒲缥温泉、施甸温泉养生洗浴等旅游项目；三岔河水库建成蓄水发电，移民搬迁安置稳步推进，境外水电开发进展顺利。

——抓改革、扩开放，不断激发新活力。党政机关公务用车、机关事业单位工作人员养老保险、殡葬改革、农村土地承包经营权确权、农村宅基地和集体建设用地使用权登记颁证等各项改革稳妥推进。腾冲成功实现撤县设市。商事制度、食品药品监管体制改革不断深化，宽进严管的市场主体诚信体系建设初见成效。腾冲边境经济合作区实体化管理迈出实质步伐，大数据管理局挂牌成立。土地流转“山邑模式”在全省推广。政务服务管理创造全省先进经验。对缅甸及印度经贸农业水电等领域合作取得新进展，密支那至班哨二级公路先期改建工程启动。

——惠民生、促和谐，不断提高人民生活水平。城乡常住居民人均可支配收入达25647元和8572元，分别增8.5%、12.4%。开发就业岗位2.65万个，贷免扶补和“两个10万元工程”培育微型企业1400户，带动1.72万人就业。各类社会保险征缴面进一步扩大。城乡困难群众基本生活得到保障。一批薄弱学校改造项目和校安工程全面完成，新增校舍21.5万平方米，滇西应用技术大学腾冲珠宝学院批准建设。疾病控制、妇幼保健、艾滋病防控、精神卫生等公共卫生和计划生育均等化服务全面落实，新农合补偿人次和补偿金分别比上年增加61万人次和1.8亿元，医疗保障水平稳步提高。深入实施精准扶贫，投入市级以上扶贫开发资金16.78亿元，实施扶贫整乡推进5个，整村推进94个，争取农发行易地搬迁专项贷款39.55亿元，减少贫困人口7万人。有效组织开展昌宁“9·16”山洪泥石流、“10·30”地震等抢险救灾。安全生产责任体系不断完善，反恐维稳处突能力进一步增强，人民群众生命财产安全得到保障，社会和谐稳定。

回顾“十二五”，面对国际国内经济下行压力和繁重的改革发展任务，全市上下攻坚克难、奋力拼搏，呈现出经济健康快速发展、基础设施水平跃升、民生不断改善、各项事业全面进步、社会和谐稳定的可喜局面，是我市历史上发展最好最快的时期之一，为全面建成小康社会奠定了坚实基础。

——经济持续较快增长，经济总量不断扩大。全市生产总值跃上500亿元新台阶，年均增长12.8%；人均生产总值年均增长12%；财政总收入和一般公共预算收入年均分别增长16.9%、19.6%；一般公共预算支出年均增长18.5%；城乡常住居民人均可支配收入年均分别增长12.3%、17.3%；社会消费品零售总额年均增长15.5%；金融机构年末存贷款余额年均分别增长17.4%、19.3%。主要经济指标保持两位数增长，增速位居全省前列。

——产业培育成效显著，经济结构明显优化。三次产业结构由“十一五”末的30.3：30.9：38.8调整为25.7：34.8：39.5，一产比重降低4.6个百分点，二产比重提高3.9个百分点，其中工业比重提高1.7个百分点。“三农”工作成绩显著，为经济社会跨越发展奠定了坚实基础，农村居民可支配收入超过全省平均水平。粮食总产连续12年增长，人均占有粮食居全省第一；两烟、畜牧、核桃、石斛、茶叶、蚕桑、咖啡、中药材、食用菌、糯橄榄、红花油茶等特色产业稳步发展，褚橙落户龙陵，完成15个全国农业标准示范区建设，数量为全省第一；高黎贡山（生态茶）、CY（永昌硅）、腾药获得中国驰名商标。认证国家地理标志证明商标20件，为全省第一；全国质量强市示范城市创建通过省级预验收；获准全国知名品牌创建示范区3个，全省第一，全国领先。园区经济发展迅猛，保山工贸园区、腾冲经济开发区被省政府认定为省级高新技术产业开发区，生物资源加工、矿冶、建材、能源等产业快速发展，工业增加值比“十一五”末增长1.2倍，年均增17.4%。民营经济快速发展，增加值占生产总值比重达49.6%，较“十一五”末提高10.9个百分点。旅游、金融、物流等服务业加快发展，接待海内外旅游者、实现旅游业总收入分别为“十一五”末的2倍和3.5倍。

——项目投资不断强化，发展条件显著改善。累计完成固定资产投资1559亿元，是“十一五”时期的3.8倍。交通、水利、能源、城镇、信息等基础设施建设全面提速。保腾、龙瑞高速及龙腾、施孟等8条二级公路建成通车，公路通车里程达13300公里，较“十一五”末增加1588公里；农村公路建设成效显著，昌宁、施甸、龙陵实现乡村公路全面硬化，建制村通畅率达87.5%，较“十一五”末提高69个百分点。大瑞铁路建设稳步推进，保山至猴桥铁路纳入省向国家重点争取开工建设项目计划。腾冲机场改扩建及航空口岸建设扎实推进，完成保山机场盖被工程，两地间“空巴通”专项产品投入运行。能源保障能力不断增强，装机容量达192.8万千瓦，比“十一五”末增93.7万千瓦；电网建设突飞猛进，建成220千伏输变电站6座、110千伏35座，在全省率先全面解决无电人口用电问题，实现全市城乡同网同价一体化管理。中缅天然气主管及隆阳支线建成投产，施甸、腾冲、龙陵支线开工建设。新建槟榔江三岔河大型水库，建成隆阳红岩等4座中型水库和4座小（一）型水库，开工建设施甸红谷田等4座中型水库、16座小型水库及隆阳西水东调、龙陵勐糯东山引水工程，实施北庙等252座病险水库除险加固，完成187公里河道整治，建成山区“五小水利”13万件，解决了62万农村居民和学校师生饮水安全问题。棚户区改造走在全省前列，地下综合管廊建设经验在全国推广，城市建成区面积80平方公里，较“十一五”末增加20平方公里，城镇化率达42%，提高12个百分点。城乡统筹力度加大，姚关、勐糯、柯街卡斯3个市级试点进展良好，新农村建设亮点频现，整合资金53亿元，实施各类新农村建设试点2990个。94个传统村落入选国家级保护名录，居全省第一。教育、卫生、文化、体育、养老等公共服务设施进一步改善，公共服务能力明显增强。

——改革开放不断深化，发展活力有效释放。投融资、国企改革、财税、教育文化、医药卫生、收入分配等改革稳步推进，集体林权、园区管理体制等专项改革走在全省前列。行政管理体制改革有序推进，完成了11个机构相关改革，取消和下放行政审批事项294项，在全省率先试点行政机关权责清单制度。国有资产监管力度加大，国有资产总额达544亿，是“十一五”末的3.8倍。新设了一批新型金融机构，融资平台建设实现新突破，融资能力显著增强，累计融资755亿元。义乌国际商贸城一期及其电子商务平台建成运营，对缅交流合作进一步深化，与印度、孟加拉等南亚国家的交往取得突破，与泛珠三角、长三角、港澳台等发达地区合作积极推进。累计实际利用外资4.6亿美元、年均增长26.5%，引进市外到位资金1582亿元、年均增长44.7%，分别是“十一五”末的3.2倍和6.5倍。

——生态建设扎实推进，环境质量持续改善。“森林保山”“生态保山”建设加快推进，绿化荒山行动、石漠化及两江四路生态恢复治理等工程顺利实施，创新开展林业“三清”行动和森林生态资源“守护神”专项整治行动，生物多样性保护得到加强，森林覆盖率达65%，较“十一五”末提高3.1个百分点。治理水土流失面积548平方公里，为“十一五”的2.3倍。五县市区无害化垃圾处理场、城市污水处理厂及市医疗废弃物处置中心建成运行，城市垃圾无害化处理率达99%、污水集中处理率达95%。万元生产总值综合能耗控制在省考核指标以内。生态建设及环境治理制度逐步健全，全民环保意识普遍提高，生态环境质量得到提升。

——社会事业全面进步，民生保障卓有成效。投入民生类财政资金589亿元，占公共财政预算支出的78%，为“十一五”的2倍多。就业和再就业工作成效显著，城镇职工、城乡居民养老、医疗等保障体系不断健全，参保率和保障水平稳步提高，社会救助保障标准年均增长15%以上。实施保障性住房8.6万套（户）、农村危房改造及抗震安居工程12万户。住房公积金管理改革不断深化，有力缓解了职工住房困难。扶贫开发成效明显，累计投入扶贫资金49亿元，贫困人口减少45.3万人，贫困发生率降至10%以内。建立健全应急救灾体系，妥善处置各种灾害事故，人民群众生命财产安全得到保障。“两基”进一步巩固提升，学前三年毛入园率、高中阶段毛入学率分别达66.8%和85.2%，分别提高31.2和27.2个百分点，高考上线率连续9年超过全省平均水平，职业教育和高等教育稳步发展。科技进步对国民经济增长的贡献率达50%，新增国家级高新技术企业29户。与发达地区医疗卫生交流合作不断深化，新农合参合率巩固提高、报销比例逐年增长，公共卫生和基本医疗服务能力水平明显提高，人口素质稳步提升。成功创建国家公共文化服务体系示范区，我市被评为全国文化体制改革先进地区，昌宁被评为全国文化先进县。积极打造“五大文化品牌”，滇西抗战纪念馆、松山大战遗址纪念园、杨善洲精神教育基地、保山历史名人堂等一批标志性文化工程建成投入使用，永子棋院建设顺利推进。成功举办纪念抗日战争暨世界反法西斯战争胜利70周年系列活动、两届史迪威公路汽车越野赛和腾冲半程马拉松赛。全民健身运动蓬勃开展，体育竞技水平稳步提升，保山籍运动员实现奥运会、残奥会金牌、奖牌“零”的突破。与此同时，统计、决策咨询、外事、人防、档案、地方志、社科、侨务、红十字会等工作不断加强；地震、水文、气象、检验检疫、海关、邮政、保险等工作取得新成效；老龄、双拥、妇女儿童、残疾人等事业协调发展。

——民主法治全面加强，社会更加和谐稳定。扎实推进法治、责任、阳光和效能政府建设，着力提升政府依法行政能力。认真执行市人大及其常委会的决议决定，自觉接受市人大及其常委会的法律监督、工作监督和市政协的民主监督，高度重视市人大代表建议和政协提案办复工作，累计办理建议370件、提案856件，办复质量进一步提高。充分听取各民主党派、工商联、无党派人士和人民团体的意见建议，政府决策科学化、民主化、信息公开化进程有力推进。审计工作全面加强，昌宁村级财务审计在全省率先实现全覆盖，市审计局被评为全国文明单位。积极推进惩治和预防腐败体系建设，政府廉政建设和反腐败工作深入开展，各级干部廉政勤政意识明显增强，作风建设取得良好成效。政风行风上线174期，解决群众反映问题650多个。“六五”普法教育通过验收，公民法律意识和法治观念明显增强；“大调解”工作格局走在全省前列，“6995”信息平台得到国家层面肯定并在全省推广。高度重视群众和信访工作，全面排查化解矛盾纠纷。民族团结进步示范区创建成效初显，少数民族地区经济社会发展加快，民族团结、宗教和谐。国防建设不断加强，军警民团结的良好局面巩固发展。安全生产工作成绩显著，食品药品监管力度加大，各类事故总量持续下降，死亡人数低于省控指标。持续推进社会治理创新，社会治安立体化防控水平不断提升，反恐防暴和打击犯罪的快速反应机制建立，妥善应对涉缅涉边等突发事件，坚决打击邪教，禁毒防艾人民战争深入开展，群众安全感满意度居全省前列。

各位代表，同志们！成绩来之不易，这是省委、省政府正确领导的结果，是市委统揽全局、科学决策的结果，是市人大、市政协加强指导监督、支持帮助的结果，是全市广大干部群众克难奋进、扎实苦干的结果。在此，我代表市人民政府，向全市各族人民，向人大代表、政协委员，向各位老领导、老同志，向各民主党派、工商联、无党派人士、各人民团体，向驻保人民解放军、武警部队官兵，向中央和省属各部门各单位，向关心支持帮助保山建设发展的朋友们致以崇高敬意和衷心的感谢！

回顾过去，我们清醒地看到，前进的道路上还存在诸多困难。主要是：综合经济实力不强，产业配套能力弱，跨越发展任务繁重；城乡、区域发展不平衡，农民增收渠道单一，脱贫攻坚任务紧迫；社会事业发展不足，财政收支矛盾突出，保障和改善民生任务艰巨；互联互通基础薄弱，对外开放程度低，外向型经济发展滞后；政府职能转变还不到位，服务意识和水平有待提高，作风建设任重道远。我们将坚持问题导向，采取有力措施加以解决。

二、勇于争先、全力跨越，再创“十三五”新辉煌

根据市委“十三五”规划建议，市人民政府编制了“十三五”规划纲要，贯彻了习近平总书记考察云南重要讲话精神及省委省政府对保山提出的发展定位和要求，贯彻了十八届五中全会、中央经济工作会议，省委九届十二次全会、省委经济工作会议，市委三届六次全会精神。

指导思想：高举中国特色社会主义伟大旗帜，全面贯彻落实党的十八大、十八届三中、四中、五中全会和省委、市委全会精神，以马列主义、毛泽东思想、邓小平理论、“三个代表”重要思想和科学发展观为指导，深入贯彻落实习近平总书记系列重要讲话精神，准确把握“四个全面”战略布局，牢固树立创新、协调、绿色、开放、共享的发展理念，全面落实省委、省政府对保山的工作要求，持续推进“六大战略”、抓实“四化五加强”工作措施，坚决打赢脱贫攻坚战，加快建设“五基地一中心”，在面向南亚东南亚辐射中心建设中先行先试、走在前列，打造全省经济发展新的增长极，争当全省产业转型升级和生态文明建设排头兵。

突出五大发展理念：坚持创新发展，强化科技人才支撑，激发创业创新活力，提高发展速度、质量和效益；坚持协调发展，统筹推进城乡、区域、经济社会发展，增强发展的全面性、整体性和均衡性；坚持绿色发展，推动发展绿色化、低碳化，森林覆盖率提高到67%，促进生态和资源优势转化为经济优势，建成全省生态文明建设示范市；坚持开放发展，构建对外开放新机制，不断增强发展动力，拓展发展领域和空间；坚持共享发展，促进教科文卫体等社会事业全面进步，推进健康保山建设，增强各族人民幸福感。

主要发展目标：生产总值年均增长11%以上，人均生产总值增长10%以上，经济总量突破千亿元大关，总体实力显著增强；经济结构进一步优化，三次产业结构调整为20:40:40；一般公共预算收入年均增长10%以上；固定资产投资年均增长20%以上，五年累计突破4000亿元；社会消费品零售总额年均增长12%以上；城乡常住居民人均可支配收入年均增长10%和12%以上；进出口总额年均增长20%以上；金融机构存贷款余额分别增长13%和15%以上；城市建成区面积达90平方公里，城镇化率达50%以上，滇西边境中心城市基本形成；价格总水平保持基本稳定；贫困县全部摘帽，按现行标准实现27.11万贫困人口全面脱贫，确保与全国全省同步全面建成小康社会。

“十三五”时期，我们将牢牢把握六个重点：

（一）全力推动主要经济指标实现两位数增长。“十三五”期间，国际环境总体相对稳定，国内经济长期向好的基本面没有改变；全省面向南亚东南亚辐射中心、民族团结进步示范区、生态文明排头兵建设有力推进，为我市跨越发展带来了新机遇；通过多年来的艰苦努力，全市经济实力明显增强，产业培育初见成效，发展基础更加扎实，发展环境更加优化，资源区位优势和发展潜力更加显现。我们将以五大发展理念引领跨越发展，以投资、产业为抓手支撑跨越发展，以转变作风、狠抓落实推动跨越发展，深化改革，扩大开放，努力保持经济两位数增长，实现经济社会发展新跨越。

（二）全力建设“五基地一中心”。着力推进农业现代化进程，稳定“滇西粮仓”，加快发展特色经作、高效林业、山地牧业、淡水渔业、开放农业，推进一二三产业融合发展。稳步提升粮油、两烟、畜牧、茶叶、咖啡、水果、蚕桑、甘蔗八大传统产业，大力发展蔬菜、核桃、石斛、红花油茶、坚果、林下产业六大生态特色优势产业，积极培育中药材、食用菌、特色养殖、糯橄榄四大新兴产业。着力加强基地建设，培育万家欢等一批龙头企业，建设一批现代农业庄园，培植销售收入5亿元以上龙头企业10户，1亿元以上“小巨人”领军企业30户。大力实施农业标准化和品牌培育工程，全力推进农产品加工业发展，打造特色农产品加工基地。面向南亚东南亚市场，积极承接产业转移，以园区为平台，加快培育生物制药、装备制造、轻纺服装等产业，形成一批产业集群，打造国际小商品加工基地。加快推进国际数据服务产业园建设，争取国家信息中心西南电子政务外网和全省电子政务两个灾备中心布局在保山。加强数据资源整合，建设人口、法人单位、自然资源和空间地理、宏观经济、信用征信五大数据库和一批行业数据库，让同样的电力因不同用途而产生更好的效益，打造数据存储灾害备份基地。全力推动多晶硅和硅基电子材料、中国建材集团大理石板材加工等项目落地建设。积极推进天然气利用试验示范区建设，发展高档陶瓷等新型材料。着力推进龙陵动力锂离子电池和新能源汽车项目建设，加快腾冲玄武岩纤维及产业化前期工作，提升建材产业，打造新材料加工基地。加快推进义乌国际商贸城（二、三期）、工贸园区物流产业园、蒲缥火车站片区、中和片区等商贸物流项目建设，发展“互联网+”，推动加工、商贸企业普及电子商务应用，优化商业网点布局，打造商贸物流基地。加强中心城市基础设施建设，加快棚户区和危房改造，有序推进老旧住宅小区综合整治，完成东河两岸截污和景观工程，推进西山生态文化环线研究并分期实施，统筹考虑中心城市用水问题，完善城市功能、强化公共服务、提升城市品位，聚力打造“园林、生态、创业、宜居”城市。积极申报创建国家创新型试点城市，拓展全国质量强市示范城市创建成果，建成高黎贡山索道，建设万亩青华海生态湿地恢复工程、万亩生态观光农业园和万亩东山生态恢复工程，不断增强产业、教育、医疗、旅游文化等支撑辐射能力，打造滇西边境中心城市。

（三）全力推动城乡协调发展。坚持以人为本、科学发展、改革创新、依法治理，统筹空间、规模、产业三大结构和规划、建设、管理三大环节，提高城市工作的全局性和系统性。加强市政基础建设，完善城镇配套服务功能，不断提升城市环境质量、人民生活质量和城市竞争力。推进腾冲城组团发展，打造“园林、生态、宜居、文明”国际旅游城市。抓好昌宁田园城市和施甸、龙陵山水风光城市建设发展。全力推进创建卫生城市、园林城市、文明城市、生态城市，争创联合国人居奖和中国人居奖。统筹城乡一体化发展，促进城乡要素平等交换、合理配置和基本公共服务均等化，推进农业转移人口市民化。加强集镇和美丽宜居乡村建设，着力打造新房新村、生态文明、宜居宜业的新农村。

（四）全力推进开放型经济发展。深化行政审批、投融资、财税、商事制度等重点领域改革，为跨越发展提供机制体制保障。争取腾冲边境经济合作区升格为国家级边境经济合作区。以缅甸、印度等南亚东南亚地区为开放指向，加快发展轻纺服装、生物资源加工、机械装备制造、跨境物流等外向型产业，推动密支那工业园区建设，努力拓展国际市场。促成保山—密支那边境交易会轮流举办，推动甘拜地口岸对等开放，提高通关便利化水平。坚持引资、引智、引技并举，不断提升招商引资质量，引进市外到位资金年均增长15%以上。着力促进民营经济加快发展，增加值占生产总值比重达56%以上。

（五）全力推进“五网”建设。精心组织“五网”建设5年大会战，为实现跨越发展提供有力保障。建设互联互通的路网。建成绕城、保泸、昌保、腾猴、腾陇、保施6条高速公路，G555隆阳至施甸一级公路，G320保山至龙陵、G219黄草坝至龙镇桥、界头至泸水、密支那至班哨4条二级公路，3800公里乡村公路，实现县县通高速、乡乡通二级路80%以上、村村通硬化路。争取开工建设瓦窑至西邑、孙足河至大田坝、昌宁至巍山、三达地至龙镇桥、龙陵半斤坝至隔界洼5条二级公路。建成大保铁路，推动保山至腾冲猴桥铁路开工。加快伊洛瓦底江中缅陆水联运通道开发，加强中缅印国际铁路前期工作，推动交通基础设施互联互通。建设广覆盖的航空网。完成腾冲机场扩建和保山机场改造提升，两个机场建成口岸机场；建成隆阳、昌宁通用机场，启动腾冲、施甸、龙陵通用机场建设；大力拓展国际国内航线。建设安全可靠的水网。建成腾冲花园等中型水库，新开工昌宁立新等7座中型水库和21座小型水库，加快3个大型灌区和腾冲地盘关中型水库等重大项目前期工作。建设区域性国际化的能源保障网。加快500千伏高压输变电工程规划建设，完善220千伏骨干电网，改造升级城乡电网。加快29座在建水电站建设，统筹境内外电源电网建设，力争建成滇西国际电力交换枢纽和清洁能源基地。建设共享高效的互联网。加快“智慧保山”建设，推进信息惠民国家试点城市创建，抓好网络基础设施建设，扩大4G网络、光纤宽带网在农村的覆盖范围。

（六）全力打赢脱贫攻坚战。以脱贫攻坚统领全市经济社会发展，牢牢把握滇西边境连片特困地区扶贫和以施甸为主的布朗族整乡整村推进整族帮扶的机遇，全面落实扶贫攻坚“军令状”，坚持易地扶贫搬迁与统筹城乡发展相结合，改善基础设施与发展特色产业相结合，区域开发与精准扶贫到村到户相结合，科技、项目、资金扶持与转移就业培训相结合，强化教育、卫生、文化等公共服务，深入实施“63686”行动计划，2017年腾冲整体脱贫，2018年全市建档立卡贫困乡、贫困村全部脱贫，隆阳、施甸、龙陵、昌宁贫困县区摘帽退出，2020年实现贫困人口同步进入小康社会。

三、真抓实干、开拓进取，实现“十三五”良好开局

2016年经济社会发展主要指标建议为：生产总值增长11%以上；固定资产投资增长20%以上；一般公共预算收入增长10%以上；社会消费品零售总额增长12%以上；进出口总额增长15%以上；城乡常住居民人均可支配收入分别增长10%和12%；金融机构存贷款余额分别增长13%和15%；城镇登记失业率控制在4%以内；万元GDP能耗下降和落后产能淘汰、居民消费价格指数、主要污染物排放总量等控制在省考核指标以内。

围绕上述目标，我们将重点抓好九个方面工作：

（一）抓好农村经济，推进农业产业化

严守488.85万亩耕地红线，加强基本农田保护和耕地质量建设。落实粮食生产行政首长责任制，加快粮食生产功能区建设，确保粮食安全。优化农业生产结构和区域布局，着力抓好农林畜烟等高原特色优势产业发展，努力提高土地产出率和劳动生产率，实现农业增加值增6%以上。

推进农业产业品牌化。以全面提高农产品质量水平和竞争力为核心，加大对现有品牌的整合力度，加快发展无公害农产品、绿色食品和有机农产品，培育做强高黎贡山、昌宁红茶叶、新寨咖啡等一批特色名牌。

推进农业产业工业化。用工业化理念发展现代农业，大力提升农业标准化、信息化和机械化水平。用工业化的生产方式改造传统农业，加强农产品加工技术创新，促进农产品初加工、精深加工及综合利用加工协调发展，提高农产品加工转化率和附加值，重点抓好粮油、畜产品、木本油料、茶叶、咖啡、中药材等特色农产品精深加工，加大对腾药、优昊等企业扶持力度。

推进农业产业规模化。完善土地流转配套政策，加大承包土地依法有序流转力度，推广施甸“山邑模式”、龙陵“褚橙模式”，完善腾冲“公平模式”，发挥多种形式农业适度规模经营的引领作用。加强基地标准化创建、农产品流通设施和市场建设，做大特色农产品基地。大力培育新型农业经营主体和新型职业农民，扶持发展万家欢、褚橙等10户土地流转规模上万亩的农业“小巨人”领军企业。新增年销售收入1亿元以上企业5户、2000万元以上企业5户、省级龙头企业5户、市级龙头企业20户。认定市级示范家庭农场30个以上，新增省级农民合作示范社5个以上。

推进农业产业庄园化。加快生态农业产业园建设，重点规划建设隆阳国家级现代农业示范区、施甸热带水果观光庄园、腾冲界头现代农业示范园、极边乌龙茶庄园、龙陵极斛庄园、昌宁万亩中药材庄园等12个现代农业示范园区，新增市级农业庄园20个。

推进农业产业生态化。坚持生态建设产业化，产业发展生态化，持续推进“森林保山”“生态保山”建设；加快农业发展方式转变，大力发展绿色生态农业，强化农业污染治理，推进农产品质量安全检测体系建设，努力实现农产品种植、加工、流通全程生态化、绿色化。

（二）抓好园区建设，推进新型工业化

加快工业经济发展。坚持做大增量与优化存量相结合，提升传统产业与培育壮大新兴产业相结合，以重点企业、重大项目为支撑，做好运行监测分析和要素保障，实现工业增加值增14%以上。

加快园区建设。坚持错位发展、突出特色，优化园区产业规划布局，加强配套设施及园中园、特色园建设。不断完善园区管理体制机制，强化投融资平台建设。5个省级园区工业增加值增16%以上。

加强工业项目建设。抓好博翔科技弹性纤维、恒丰年产15万锭高端新型纤维纺纱、品斛堂石斛系列产品开发等重点项目建设，年内建成投产。加快龙陵动力锂离子电池、复烤厂易地技术改造、腾冲30万吨硅藻土深加工等续建项目建设。推进昌宁核桃壳高密度板等项目前期工作，争取开工。

加快民营经济发展。全面落实民营经济发展政策措施，激发市场主体创业积极性。深入实施“两个10万元”微型企业培育工程，培育壮大龙头企业，加强企业家队伍建设，促进建立现代企业制度，民营经济增加值占生产总值比重提高2个百分点。切实做好规模企业培育，新增25户以上。

加快企业技术创新。强化众创空间建设，努力营造有利于众创、众包、众扶、众筹的良好环境。不断完善科技创新体制机制，大力加强人才队伍建设。组织实施一批重大科技项目，突破制约产业发展的关键技术；加大对科技型中小企业的扶持力度，促进产业技术创新，争取新认定一批高新技术企业、创新型企业和企业技术中心。

加快信息化建设。以IDC产业布局为核心，加快推进国际数据服务产业园与数据库建设，实现强政惠民兴企。加快建设电子政务异地灾备中心，同步推进网络与信息安全技术保障设施建设。深入实施“宽带乡村”等通信工程，全面推进信息惠民国家试点城市创建。

（三）抓好服务业，推进第三产业提质增效

加快发展现代服务业。切实抓好城乡市场建设和龙头企业培育，巩固提升交通运输、批发零售、住宿餐饮等传统服务业，落实好国家促进消费政策措施。加快电子商务发展，建设义乌商贸城、水长园区和腾冲3个市级电子商务示范产业园，推进“义乌购”“云贡城”“保山邻里”等平台建设，支持企业依托第三方电商平台开展网上销售。大力发展农村电商，提升农特产品网络销售能力。积极培育养老健康家政、节能环保、文化教育体育等服务消费，强化农村消费市场建设，激发农村消费活力。发展普惠金融，增设银行业金融机构，加强风险防控，建立与现代产业发展相适应的金融体系。积极培育对外贸易增长点，优化进出口商品结构，促进外贸平稳增长。实现服务业增加值增11%以上。

加快发展旅游产业。全力打造“三张名片”，制定实施“旅游+”行动计划，启动高黎贡山国家公园、温泉国家公园、怒江大峡谷旅游综合体等重点项目建设，建成境内高速公路及主要干线自驾游标识牌，打造隆阳滇西自驾大本营。大力发展乡村旅游，完成第一批20个、启动第二批15个特色旅游村建设。积极发展红色旅游，重点打造杨善洲干部教育学院、松山大战纪念园、腾冲抗战文化旅游环线。加快发展温泉度假、养老养生、户外运动和跨境旅游。提升旅游信息化水平，实现旅游服务全面上线，主要景区景点免费WIFI全覆盖。努力发展航空旅游，开通保山至杭州、深圳和腾冲至上海航线。进一步规范旅游市场，维护游客和经营者合法权益。接待海内外旅游者1410万人次，实现旅游总收入135.5亿元。

（四）抓好基础设施建设，推进投资持续增长

推进“四个一百”重点项目建设。不断完善投资工作责任制、项目建设督查制，强化要素保障和协调服务，破解项目实施中的难题，实现固定资产投资增20%以上。

推进综合交通建设。确保龙江特大桥5月通车，加快建设保泸高速、G320线保山境内段、大瑞铁路、密支那至班哨二级公路先期改建工程，开工建设昌保高速、腾冲机场连接线高速、黄草坝至龙镇桥二级公路。抓好腾猴、腾陇、保施等高速公路前期工作。加快推进“四好农村公路”建设，提前完成建制村通畅工程；抓好施甸、昌宁、龙陵等城乡客运站建设。加快腾冲机场改扩建工程进度，启动保山机场改造提升工程，开工建设昌宁通用机场，抓实隆阳、施甸、腾冲、龙陵通用机场前期工作。

推进农田水利工程建设。加快在建的4座中型、12座小型水库及2件调水工程建设，开工建设龙陵轱辘河等一批中小型水库，启动实施农村饮水安全巩固与提升工程，开工建设一批抗旱水源、河道治理、中低产田地改造等工程。

推进能源建设。继续加强电网建设，加快槟榔江三岔河水库、龙川江水库及电站等项目，抓好移民搬迁安置，推进怒江流域水电开发前期工作。加快施甸、腾冲、龙陵天然气管网支线建设，抓好中心城区及园区企业“煤改气”工作，提高天然气利用率。

鼓励和扩大民间投资。大力推行PPP模式，加快一批市政基础设施、公用事业项目建设，努力提高民间投资占固定资产投资比重。

（五）抓好城乡建设，推进区域协调发展

加强城乡规划工作。促进“多规合一”，提升规划水平，严格规划管理，增强规划的公开性和权威性。尽快完成隆阳、施甸、昌宁等坝区村镇体系规划和村庄规划，实现市县乡村四级规划全覆盖。

强化城镇基础设施建设。进一步深化城镇建设投融资体制改革，加大市政交通、能源通信、给排水、电力燃气、污水和垃圾处理等基础设施投资，统筹教育、文化、卫生、体育、专业市场等公共服务配套，增强城镇的综合承载能力。坚持产城融合，统筹发展物流、金融、商贸、旅游等现代服务业。积极推进智慧城市、海绵城市建设，加快完善北城区、东城区、青阳片区和板桥、辛街功能片区基础设施。全面加快棚户区改造、地下综合管廊等建设。持续打造一批重点特色小镇。促进房地产业健康发展，实现建筑业增加值增长16%，城镇化率达43.6%。

加快美丽宜居乡村建设。按照名镇名村和传统村落建设要求，启动一批传统村落保护项目。坚持连片扶贫开发整乡推进与美丽家园、特色旅游小镇建设相结合，以中心村、特色村和传统村落为重点，打造300个美丽宜居乡村建设示范点。扎实抓好隆阳区、腾冲市两个省级统筹城乡试点，推进板桥镇国家级新型城镇化试点及3个市级试点实现新突破。深化户籍制度改革，完善农业转移人口权益保障政策，推进农业转移人口市民化。

提高城市治理能力。完善城市治理体系，落实主体责任，创新管理模式，推进网格化管理和平安社区创建，强化综合执法、文明执法，提高城市管理服务水平。全面启动城市“四创两争”工作，隆阳、腾冲达到国家卫生城市标准。着力解决农贸市场、公厕等设施布局不合理问题，推进中心城市公交改革发展，加强对城市交通、噪声污染、建筑扬尘、占道经营、环境卫生等专项整治，着力提升人居环境。

（六）抓好改革开放，推进发展动力活力提升

深化重点领域改革。继续精简行政审批事项，加大政府购买公共服务力度，深化事业单位分类改革。按照省政府批复要求完成公务用车制度改革任务。积极推进供给侧结构性改革，提高供给体系质量和效率。扎实开展农村土地承包经营权确权登记颁证工作，完善试点、全市推开。加大农村土地流转力度，稳妥推进土地经营权、林地使用权和农民财产权确权抵押担保转让等改革。扎实开展不动产统一登记工作。加快沿边金融综合改革试验区建设，推动地方新型金融组织创新发展，加大对县市区融资支持力度。深化商事制度改革，强化市场信用体系及监管体系建设，降低企业成本。深入推进财税改革，加强财政专项资金清理整合、绩效评价和政府债务管理，提高财政资金使用效率。加快殡葬改革步伐，积极推行绿色文明殡葬。加快教育综合改革，统筹城乡义务教育资源均衡配置。抓好医疗卫生综合改革和公共文化服务标准化试点改革，提升服务能力和水平。

扩大沿边开放。深化与缅甸、印度、孟加拉交流合作，力争在农业、经贸、交通、旅游、能源、跨境金融等领域实现新突破。落实外贸发展政策措施，促进人员、物资、货币流动通畅，进出口总额增15%以上。围绕重大项目建设、重点产业发展，着力引进一批带动力强的大项目大企业，引进市外到位资金增20%以上。

（七）抓好民生改善，推进社会事业全面进步

抓实脱贫攻坚。按照“五个一批”要求，拓展脱贫路径，着力抓好精准脱贫。投入各类扶贫开发资金30亿元，扎实开展易地搬迁、产业扶贫、社会帮扶等各项工作，强化“挂包帮”“转走访”，着力解决贫困地区群众出行、饮水、居住、就医、上学等困难，确保5个建档立卡贫困乡出列、72个贫困村退出、6万人脱贫。

促进就业创业。统筹解决好大中专毕业生、下岗失业职工、失地农民、退转军人等重点人群的就业问题。认真落实“大众创业，万众创新”政策，构建城乡均等的公共创业就业服务体系，推动发展“互联网+”创业服务，以创业带动就业。充分发挥创业贷款促进就业作用，激发大众创业热情。加强就业培训和职业能力建设，做好就业服务指导，为用工单位和求职人员提供便捷高效的服务。

提高社会保障水平。进一步扩大养老、医疗、失业、工伤、生育保险覆盖面，健全完善社会救助体系，提高社会保障水平。加强残疾人保障和服务体系建设，促进残疾人事业健康发展。积极开展应对人口老龄化行动，强化城乡养老服务设施建设，鼓励支持社会资本兴办养老服务机构。完成昌宁地震、泥石流灾后恢复重建。推进自然灾害综合防治，加强应急救援力量建设，提高应急保障能力。

坚持教育优先发展。加强学校基础设施建设，全面落实教育惠民政策，促进教育公平。大力发展学前教育，加快发展高中阶段教育，巩固提升义务教育，统筹抓好民办教育、特殊教育、老年人教育。加强“双师型”教师队伍和实训基地建设，提升教师队伍素质能力，创新教育管理模式，探索城乡教师交流机制。坚持产教融合，推进职业教育园区建设，加快现代职业教育和应用技术教育发展，努力培养产业技术工人。支持保山学院向应用技术转型发展，推动保山医专升本，提升办学能力和水平，抓好珠宝学院建设和招生工作，积极申报其他特色学院。

强化公共卫生、医疗服务。加快东城区市人民医院迁建项目和乡镇卫生院、社区卫生服务机构、村卫生室标准化建设。继续深化医疗卫生滇沪合作，提升服务水平。继续完善新农合制度，巩固基本用药制度，健全农村医疗保障体系，巩固参合率，推进全国基层中医药先进市创建。实施“关爱妇女儿童健康行动”，提高健康水平。推动市县乡村医院、卫生院信息联网。全面落实一对夫妇可生育两个孩子政策，促进人口均衡发展。

发展文化体育事业。加大文化基础设施建设力度，巩固公共文化服务体系示范区创建成果。挖掘弘扬优秀传统文化，加强文化遗产保护和开发利用。扩大文化对外交流。广泛开展群众文化活动，倡导全民阅读，鼓励文艺创作，打造一批文艺精品，提升广播电视服务水平，推动文化事业和文化产业协调发展。加强城乡公共体育设施建设，推进“七彩云南全民健身工程”，指导开展好群众性体育活动，办好第四届市运会，推动体育事业和产业健康发展。

加强食药品安全监管能力和应急体系建设，确保广大人民群众饮食用药安全。认真组织开展第三次农业普查，摸清“三农”家底。统筹推进决策咨询、社科、史志、人防、侨务、红十字、档案等工作，促进各项事业健康发展。

（八）抓好民主法治建设，推进社会和谐稳定

自觉接受市人大及其常委会的法律监督和工作监督，主动接受市政协的民主监督，重视公众监督，广泛听取各民主党派、工商联、无党派人士的意见和建议，积极支持工会、共青团、妇联等人民团体的工作。全面推进依法治市，开展中心城市管理、昌宁田园风光城市保护等地方立法工作，启动“七五”普法教育，提高全民法律素质和法治意识。继续推进社会主义精神文明建设，培育和践行社会主义核心价值观。认真贯彻落实党的民族和宗教政策，深入推进民族团结进步示范区建设，进一步促进民族团结、宗教和睦。更加重视国防教育，加强民兵预备役部队建设，积极开展拥军优属和军民共建活动，推动军民深度融合发展。抓好群众和信访工作，建立健全群众利益诉求表达机制、强化重大决策社会风险评估，有效预防和化解社会矛盾。加强城乡和谐社区建设，抓好村（社区）换届工作。加快推进现代社会组织体系建设，充分发挥社会组织在加强和创新社会治理中的积极作用。健全细化安全生产“党政同责、一岗双责、失职追责”的责任体系，严控安全生产重大事故发生，保障人民群众生命财产安全。以“法治保山”为抓手，深化“6995”信息平台建设，健全反恐和社会治安防控体系，做好防范和处理邪教工作，依法打击各种犯罪行为，创建最安全城市，维护社会和谐稳定。

（九）抓好政府自身建设，推进政府职能转变

实现跨越发展目标，对政府工作提出了新的更高的要求。我们将深入贯彻市委“闯”字当头、“实”字在先、“干”字为要的精神，把心思集中到“想”干事上，把本领体现在“会干事”上，把目标锁定在“干成事”上，坚持落实第一，不断提升执行力。进一步深化简政放权、放管结合，优化服务，加快政府职能转变，不断提升依法行政水平。深入开展“两学一做”学习教育，发挥领导干部这个“关键少数”的带动作用，争做跨越发展先锋。巩固“三严三实”和“忠诚干净担当”专题教育成果，推进作风建设常态化，坚决防止“四风”反弹回潮。压实部门责任担当，加强履职服务情况监督检查，坚决整治不作为、乱作为，提高政府效能。推进审计制度改革，加强对公共资金、国有资产、公共资源和领导干部履行经济责任情况的审计监督。强化党风廉政建设，加大权力运行制约和监督力度，努力建设清廉政府。

今年，市政府将突出办好8件实事：1.实施整乡推进5个，易地扶贫搬迁1万户、3.5万人，确保6万人脱贫；2.实施棚户区改造8517套（户），完成农村危房改造和抗震安居工程建设任务2.5万户；3.打好禁毒人民战争，严厉打击涉毒违法犯罪，加强禁吸戒毒，严控吸毒人员增长，建成腾冲、龙陵、昌宁戒毒康复中心；4 .建成1个市级和2个县级电子商务服务中心、4个乡镇电子商务服务站，培训人员1万人以上，电子商务交易额25亿元，增长50%；5.加快中心城市“一院三校”建设，确保市人民医院迁建项目一期具备搬迁条件，“三校”年内实施整体搬迁；6.投资16.77亿元，抓实农村电网改造升级工程，使农村电网水平走在全省前列。7.“6995”升格为“995”政务管理平台，重点解决“三留人员”关爱服务问题，并覆盖重点乡镇；8.实现6万农村人口饮水安全提质增效。

各位代表！实现保山跨越发展，是全市人民的共同期盼，是我们的光荣使命和责任担当。让我们紧密团结在以习近平同志为核心的党中央周围，在省委、省政府和市委的坚强领导下，在市人大、市政协的监督和支持下，团结拼搏，继往开来，为全面建成小康社会宏伟目标而努力奋斗！
